# Neutralizing antibody responses in patients hospitalized with SARS-CoV-2 Delta or Omicron infection

**DOI:** 10.1172/JCI164303

**Published:** 2022-12-01

**Authors:** Susanne L. Linderman, Lilin Lai, Estefany L. Bocangel Gamarra, Max S.Y. Lau, Srilatha Edupuganti, Diya Surie, Mark W. Tenforde, James D. Chappell, Nicholas M. Mohr, Kevin W. Gibbs, Jay S. Steingrub, Matthew C. Exline, Nathan I. Shapiro, Anne E. Frosch, Nida Qadir, Meredith E. Davis-Gardner, M. Juliana McElrath, Adam S. Lauring, Mehul S. Suthar, Manish M. Patel, Wesley H. Self, Rafi Ahmed

**Affiliations:** 1 Emory Vaccine Center,; 2 Department of Microbiology and Immunology,; 3 Department of Pediatrics,; 4 Department of Biostatistics and Bioinformatics, and; 5 The Hope Clinic, Emory University, Atlanta, Georgia, USA.; 6 CDC, Atlanta, Georgia, USA.; 7 Department of Pediatrics, Vanderbilt University Medical Center, Nashville, Tennessee, USA.; 8 Department of Emergency Medicine, University of Iowa, Iowa City, Iowa, USA.; 9 Department of Medicine, Wake Forest School of Medicine, Winston-Salem, North Carolina, USA.; 10 Department of Medicine, Baystate Medical Center, Springfield, Massachusetts, USA.; 11 Department of Medicine, The Ohio State University, Columbus, Ohio, USA.; 12 Department of Emergency Medicine, Beth Israel Deaconess Medical Center, Boston, Massachusetts, USA.; 13 Department of Medicine, Hennepin County Medical Center, Minneapolis, Minnesota, USA.; 14 Department of Medicine, UCLA, Los Angeles, California, USA.; 15 Vaccine and Infectious Disease Division, Fred Hutchinson Cancer Research Center, Seattle, Washington, USA.; 16 Departments of Medicine and of Microbiology and Immunology, University of Michigan, Ann Arbor, Michigan, USA.; 17 Department of Emergency Medicine and Vanderbilt Institute for Clinical and Translational Research, Vanderbilt University Medical Center, Nashville, Tennessee, USA.

**Keywords:** COVID-19, Immunoglobulins

**To the Editor:** Humoral and cellular immune responses contribute to overall protective immunity against SARS-CoV-2, with neutralizing antibody playing a key role in preventing viral infection. This is evident from the large number of Omicron infections occurring in vaccinated and convalescent patients, since antibodies induced after vaccination or infection by the ancestral WA1 strain do not neutralize Omicron variants efficiently (1, 2). These findings have led to the FDA recommendation for inclusion of the Omicron variant in bivalent COVID-19 vaccines. However, issues have been raised about the value of adding Omicron to the vaccine based on data showing only modest differences between antibody responses after booster immunization with Omicron- versus WA1-based vaccines (3, 4). Also, a recent study has shown that booster responses to Omicron infection are affected by previous SARS-CoV-2 infections (5). Thus, having additional information on the types of neutralizing antibody responses induced after infection with different SARS-CoV-2 variants will be helpful in addressing this important issue.

Here we report live virus neutralization titers against WA1 and the Delta and Omicron (lineages BA.1, BA.2, and BA.5) variants in serum samples collected from hospitalized patients infected with SARS-CoV-2 Delta or Omicron strains. Blood samples were collected from 187 patients hospitalized with acute COVID-19 in the period from July 2021 to March 2022 at 8 US hospitals (Supplemental Methods; supplemental material available online with this article; https://doi.org/10.1172/JCI164303DS1). Of these patients, 26% were immunocompromised (details are shown in Supplemental Table 1). The majority (69%) of the patients were sequence-confirmed for Delta or Omicron infection, and the remaining were classified according to the variant that was dominant at the time of infection. Patients were unvaccinated (*n* = 80) or vaccinated with COVID-19 mRNA (*n* = 100) or adenovirus vector (*n* = 7) vaccine before infection.

In unvaccinated Delta-infected patients, the neutralizing antibody response was highly biased toward the infecting Delta strain, with lower titers against WA1 (6-fold) and strikingly lower titers against BA.1 (60-fold) and BA.2 (22-fold) (Figure 1A). In vaccinated Delta-infected patients, neutralization titers against Delta and WA1 were similar, but again were much lower against BA.1 (17-fold) and BA.2 (9-fold) (Figure 1B). Thus, both unvaccinated and vaccinated Delta-infected patients had significantly lower neutralizing antibody responses to Omicron (as determined by Wilcoxon’s rank sum test). A strikingly different pattern was seen in Omicron-infected patients irrespective of their vaccination history, with a more favorable neutralizing antibody response to BA.1 and BA.2. While BA.1- and BA.2-neutralizing titers were modestly lower in vaccinated compared with unvaccinated Omicron-infected patients, there was a clear trend toward a broader and more balanced antibody response, with similar neutralization titers in response to Delta, BA.1, and BA.2 (Figure 1, C and D). Importantly, the ratio of BA.1- to WA1-neutralizing titers was significantly higher in Omicron- compared with Delta-infected patients in both the unvaccinated (19-fold) and vaccinated (11-fold) groups (Figure 1, E and F). This analysis of the ratio of neutralizing antibody between Omicron versus WA1 within a given individual shows that Omicron infection elicits Omicron-specific antibody responses. These Omicron-neutralizing antibodies could have emerged from either de novo naive B cells or cross-reactive memory B cells.

Given the current dominance of the BA.5 strain, we then tested neutralization against BA.5. Note that our samples were collected prior to the dominance of BA.5, and most of the Omicron-infected patients in our study had been infected with BA.1 (Supplemental Table 1). In the subset of Omicron patient samples that we analyzed, there was detectable neutralization of BA.5, but it was lower than for BA.1. and BA.2 in both vaccinated and unvaccinated patients (Figure 1, G and H, and Supplemental Figure 1). This reduced neutralization activity against BA.5 in patients infected with BA.1 suggests that it is better to have BA.5 than BA.1 in the vaccine.

In summary, our results show that Omicron infection of unvaccinated or vaccinated patients induces a more proportional and balanced neutralizing antibody response to Omicron variants, supporting the recent decision to include Omicron in the bivalent SARS-CoV-2 vaccine. However, it remains to be seen how these findings from infection will translate to vaccination. Our results are from hospitalized patients with high levels of infected cells and antigen that would have efficiently induced a primary response to Omicron in addition to selectively recruiting Omicron-reactive memory B cells. It is possible that a single immunization with the Omicron bivalent vaccine may not be sufficient and that 2 doses may be needed to achieve the desired antibody response (6). Future studies should address this issue, and it will also be interesting to see how the bivalent vaccine works in people who have already been infected with an Omicron strain.

## Supplementary Material

Supplemental data

## Figures and Tables

**Figure 1:**
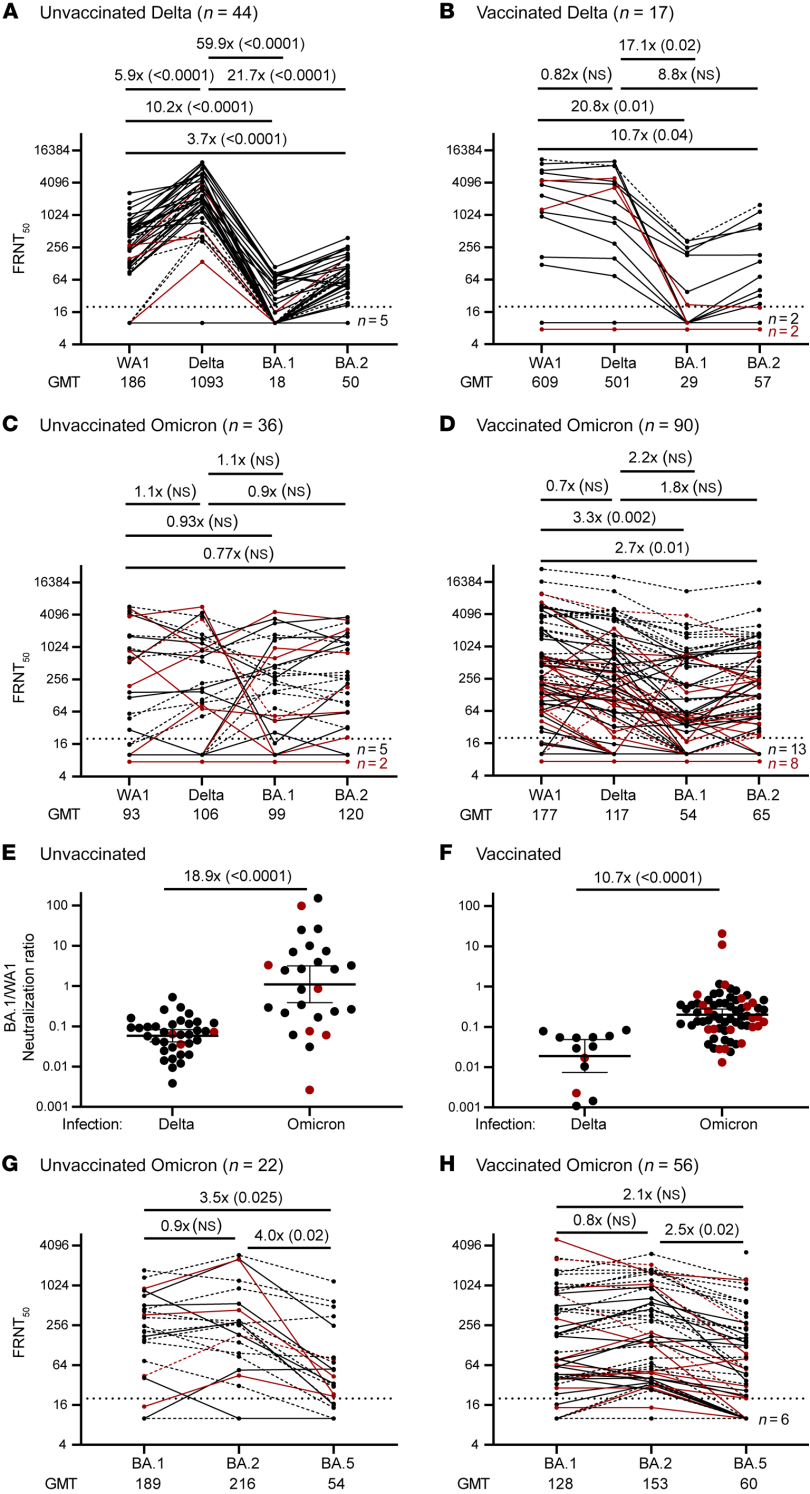
Live virus–neutralizing antibody titers in hospitalized patients infected with SARS-CoV-2 Delta or Omicron variants. In vitro neutralization titers against live WA1, Delta, BA.1, or BA.2 variants in unvaccinated (**A** and **C**) and vaccinated (**B** and **D**) hospitalized patients infected with a Delta (**A** and **B**) or Omicron (**C** and **D**) strain. Solid lines indicate sequence-confirmed infections; dashed lines, non-sequence-confirmed; red indicates immunocompromised patients. The ratio of BA.1- to WA1-neutralizing titers in unvaccinated (**E**) or vaccinated (**F**) patients. Geometric mean and 95% CI are indicated. For a subset of Omicron-infected patients who were unvaccinated (**G**) or vaccinated (**H**), BA.5 neutralization was also assessed. Values above the panels indicate fold differences between groups (*P* value). *P* values were calculated by Wilcoxon’s rank sum test.

## Version 1 10/18/2022

In-Press Preview

## Version 2 12/01/2022

Electronic publication

## References

[1] Altarawneh HN (2022). Protection against the Omicron variant from previous SARS-CoV-2 infection. N Engl J Med.

[2] Pajon R (2022). SARS-CoV-2 Omicron variant neutralization after mRNA-1273 booster vaccination. N Engl J Med.

[3] Gagne M (2022). mRNA-1273 or mRNA-Omicron boost in vaccinated macaques elicits similar B cell expansion, neutralizing responses, and protection from Omicron. Cell.

[4] https://www.statnews.com/2022/06/29/fda-dont-rush-to-change-covid-19-vaccine-composition/.

[5] Reynolds CJ (2022). Immune boosting by B.1.1.529 (Omicron) depends on previous SARS-CoV-2 exposure. Science.

[6] Ellebedy AH (2020). Adjuvanted H5N1 influenza vaccine enhances both cross-reactive memory B cell and strain-specific naive B cell responses in humans. Proc Natl Acad Sci U S A.

