# Supplementary material for: Neutralizing antibody responses in patients hospitalized with SARS-CoV-2 Delta or Omicron infection
Source: J Clin Invest. 2022 Dec 1;132(23):e164303. doi: 10.1172/JCI164303 (PMC9711871; doi:10.1172/JCI164303)
Supplement: Supplemental data [file jci-132-164303-s162.pdf]

Neutralizing Antibody to Omicron BA.1, BA.2 and BA.5 in COVID-19 Patients

Supplemental Materials

Supplemental Methods …………………………………………………………………………………………..……..2

Supplemental Figure 1. Live virus neutralizing antibody titers in hospitalized patients infected with SARS-CoV-2 Delta or Omicron variants ………………………………………………………………………..…………...5

Supplemental Table 1. Demographic and clinical cohort characteristics of adult inpatients hospitalized with COVID-19 who provided serum for analysis ………………………………………………………………….…6

References ……………………………………………………………………………………..……..………..7

Supplemental Methods

Surveillance

This prospective, multicenter observational assessment was conducted by the Influenza and Other Viruses in the Acutely Ill (IVY) Network in collaboration with the US Centers for Disease Control and Prevention (CDC). The IVY Network is a 21-hospital collaborative in the US studying COVID-19. Eight hospitals participated in the serology component included in the current analysis. The current analysis included adult patients hospitalized at IVY sites during the period of July 4, 2021–March 30, 2022. Site personnel prospectively identified and enrolled hospitalized COVID-19 case-patients. Participants were adults (≥18 years old) admitted to the hospital with symptomatic COVID-19 confirmed with a positive SARS-CoV-2 reverse transcription-polymerase chain reaction (RT-PCR) test within 14 days of symptom onset. Participants had one or more of the following COVID-19-associated signs or symptoms: fever, cough, shortness of breath, loss of taste, loss of smell, use of respiratory support (high flow oxygen by nasal cannula, non-invasive ventilation, or invasive mechanical ventilation) for the acute illness, or new pulmonary findings on chest imaging indicating pneumonia. For confirmation of SARS-CoV-2 infection, nasal specimens were centrally tested at Vanderbilt University Medical Center by RT-qPCR for SARS-CoV-2 nucleocapsid 1 and nucleocapsid 2 targets using CDC designed primers and probes. Specimens positive for SARS-CoV-2 were sent to University of Michigan for viral whole genome sequencing as described below. For respiratory specimens that could not be sequenced, periods of predominant circulation for Delta, and Omicron were defined based on time windows when each variant was identified in more than 50% of cases successfully sequenced in the study— Delta period: 4 July to 25 December 2021; Omicron period: 26 December 2021 to March 30, 2022. Trained personnel at enrolling sites collected patient data on demographics, medical history, underlying health conditions, COVID-19 vaccination status, and clinical outcomes through patient or proxy interviews and medical record review. A convenience sample of patients had a serum specimen during hospitalization. These activities were reviewed by CDC, were conducted consistent with applicable federal law and CDC policy (45 C.F.R. part 46.102(l)(2), 21 C.F.R. part 56; 42 U.S.C. §241(d); 5 U.S.C. §552a; 44 U.S.C. §3501 et seq), and were determined to be public health surveillance with waiver of informed consent by institutional review boards at CDC and each enrolling site.

Molecular diagnosis and sequencing

Upper respiratory specimens with detection of either N1 or N2 with a cycle threshold ≤32 were shipped to the University of Michigan (Ann Arbor, Michigan) for viral whole genome sequencing using the ARTIC Network protocol (v4.1 primer set) and an Oxford Nanopore Technologies GridION instrument.

Viruses and cells

VeroE6-TMPRSS2 cells were generated and cultured as previously described(1). nCoV/USA_WA1/2020 (WA/1), closely resembling the original Wuhan strain was propagated from an infectious SARS-CoV-2 clone as previously described(2). icSARS-CoV-2 was passaged once to generate a working stock. The B.1.617.2, BA.1 and BA.2 variants were isolated and propagated as previously described (1, 3). The BA.5 isolate was kindly provided by Dr. Richard Webby (St Jude Children's Research Hospital) was plaque purified and propagated once in VeroE6-TMPRSS2 cells to generate a working stock. All viruses used in this study were deep sequenced and confirmed as previously described (1).

Focus Reduction Neutralization Test

FRNT assays were performed as previously described (1, 4, 5). Briefly, samples were diluted at 3-fold in 8 serial dilutions using DMEM (VWR, #45000-304) in duplicates with an initial dilution of 1:10 in a total volume of 60 μl. Serially diluted samples were incubated with an equal volume of WA1/2020, B.1.617.2 (Delta), or BA.1, BA.2 or BA.5 (Omicron) with 100-200 foci per well at 37° C for 1 hour in a 96-well plate. The antibody-virus mixture was then added to VeroE6-TMPRSS2 cells and incubated at 37°C for 1 hour. Post-incubation, the antibody-virus mixture was removed and 100 μl of pre-warmed 0.85% methylcellulose overlay was added to each well. Plates were incubated at 37° C for 18 to 40 hours and the methylcellulose overlay was removed and washed six times with PBS. Cells were fixed with 2% paraformaldehyde in PBS for 30 minutes. Following fixation, plates were washed twice with PBS and permeabilization buffer (0.1% BSA, 0.1% Saponin in PBS) was added to permeabilized cells for at least 20 minutes. Cells were incubated with an anti-SARS-CoV spike primary antibody directly conjugated to Alexaflour-647 (CR3022-AF647) overnight at 4°C. Cells were then washed twice with 1x PBS and imaged on an ELISPOT reader (CTL Analyzer).

Quantification and Statistical Analysis

Antibody neutralization was quantified by counting the number of foci for each sample using the Viridot program (6). The neutralization titers were calculated as follows: 1 - (ratio of the mean number of foci in the presence of sera and foci at the highest dilution of respective sera sample). Each specimen was tested in duplicate. The FRNT-50 titers were interpolated using a 4-parameter nonlinear regression in GraphPad Prism 9.2.0. Samples that do not neutralize at the limit of detection at 50% are plotted at 20 and was used for geometric mean and fold-change calculations. The Wilcoxon rank sum test was used for testing differences between groups. Whenever applicable, corrections for multiple testing were performed using the p-value adjustment method by Holm (7).

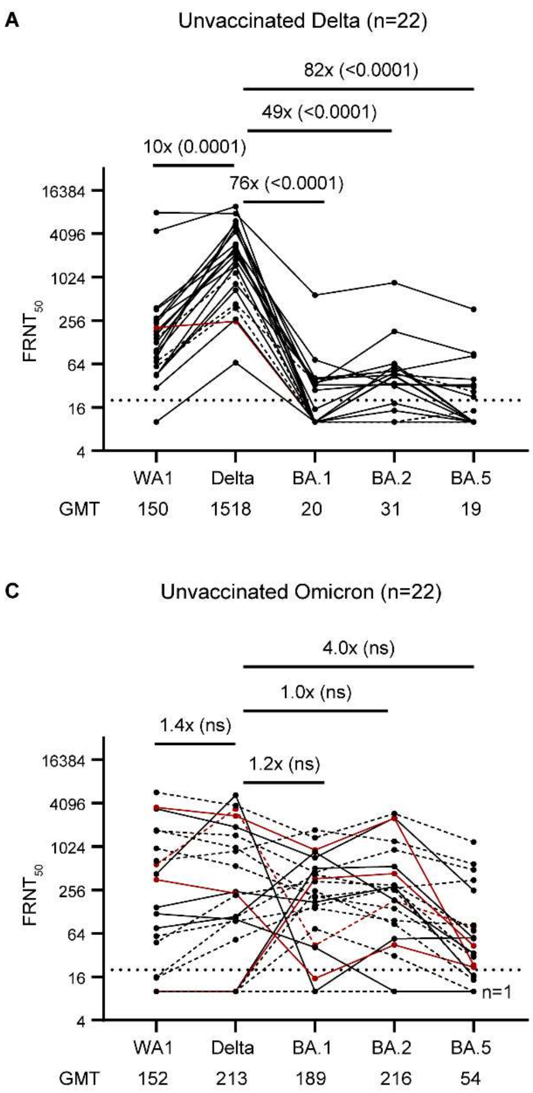

Supplemental Figure 1. Live virus neutralizing antibody titers in hospitalized patients infected with SARS-CoV-2 Delta or Omicron variants. In vitro neutralization titers ($FRNT_{50}$ or focus reduction neutralization test) against live WA1, Delta, BA.1, BA.2, or BA.5 virus in unvaccinated (A) and vaccinated (B) hospitalized patients infected with a Delta strain. Geometric mean titers (GMT) are listed below each panel. The limit of detection of 20 is indicated by a dotted line. In vitro neutralization titers against live WA1, Delta, BA.1, BA.2, or BA.5 virus in unvaccinated (C) and vaccinated (D) hospitalized patients infected with an Omicron strain. Solid connecting lines indicate samples from patients with sequence confirmed infection, while dashed lines indicate samples that were not sequence confirmed but assigned to a group due to the prevalence of contemporary strains. Red lines indicate that the patient had an immunocompromising condition or treatment. All p values found in parenthesis were calculated by Wilcoxon rank sum test.

Supplemental Table 1. Demographic and clinical cohort characteristics of adult inpatients hospitalized with COVID-19 who provided serum for analysis

	Unvaccinated Delta (n=44)	Vaccinated Delta (n=17)	Unvaccinated Omicron (n=36)	Vaccinated Omicron (n=90)
**Female Sex (%)**	13 (29.5)	4 (23.5)	18 (50.0)	39 (43.3)
**Mean Age, Years (SD)**	49.0 (13.5)	66.5 (15.6)	55.8 (17.3)	62.0 (16.1)
**Sequence confirmed (%)**	36 (81.8)	16 (94.1)	22 (61.1)	55 (61.1)
**Immunocompromised (%)**	4 (9.1)	4 (23.5)	10 (27.7)	30 (33.3)
**Solid organ transplant treated with immunosuppressive medication**		3	4	12
**Solid organ cancer treated with immunosuppressive medication**	1		1	4
**Hematologic cancer treated with immunosuppressive medication**	2		3	8
**Autoimmune disease treated with immunosuppressive medication**	1	1	1	3
**Other diseases treated with immunosuppressive medication**			1	2
**HIV**				1
**ICU (%)**	33 (75.0)	10 (58.8)	14 (51.9)	20 (27.8)
**Days since symptom onset (SD)**	17.9 (13.4)	7.9 (7.3)	9.3 (19.1)	5.9 (17.7)
**Days since last vaccine dose to hospitalization (SD)**	-	160.1 (50.8)	-	150.6 (107.1)
**# of mRNA vaccine doses (%)**				
**1**	-	1 (5.9)	-	8 (8.9)
**2**	-	16 (94.1)	-	46 (51.1)
**3**	-	-	-	26 (28.9)
**4**	-	-	-	2 (2.2)
**5**	-	-	-	1 (1.1)
**# of adenovirus vaccine doses (%)**				
**1**	-	-	-	6 (6.7)
**2**	-	-	-	1 (1.1)
**Omicron Sequences (%)**				
**B.1.1.529**	-	-	7 (19.4)	24 (26.7)
**BA.1**	-	-	2 (5.6)	5 (5.6)
**BA.1.1**	-	-	11 (30.6)	18 (20.0)
**BA.2**	-	-	1 (2.8)	6 (6.7)
**BA.2.1**	-	-	1 (2.8)	-

References

1. Edara VV, Pinsky BA, Suthar MS, Lai L, Davis-Gardner ME, Floyd K, et al. Infection and Vaccine-Induced Neutralizing-Antibody Responses to the SARS-CoV-2 B.1.617 Variants. *N Engl J Med.* 2021;385(7):664-6.
2. Xie X, Muruato A, Lokugamage KG, Narayanan K, Zhang X, Zou J, et al. An Infectious cDNA Clone of SARS-CoV-2. *Cell Host Microbe.* 2020;27(5):841-8 e3.
3. Edara VV, Manning KE, Ellis M, Lai L, Moore KM, Foster SL, et al. mRNA-1273 and BNT162b2 mRNA vaccines have reduced neutralizing activity against the SARS-CoV-2 omicron variant. *Cell Rep Med.* 2022;3(2):100529.
4. Vanderheiden A, Edara VV, Floyd K, Kauffman RC, Mantus G, Anderson E, et al. Development of a Rapid Focus Reduction Neutralization Test Assay for Measuring SARS-CoV-2 Neutralizing Antibodies. *Curr Protoc Immunol.* 2020;131(1):e116.
5. Edara VV, Norwood C, Floyd K, Lai L, Davis-Gardner ME, Hudson WH, et al. Infection- and vaccine-induced antibody binding and neutralization of the B.1.351 SARS-CoV-2 variant. *Cell Host Microbe.* 2021;29(4):516-21 e3.
6. Katzelnick LC, Coello Escoto A, McElvany BD, Chavez C, Salje H, Luo W, et al. Viridot: An automated virus plaque (immunofocus) counter for the measurement of serological neutralizing responses with application to dengue virus. *PLoS Negl Trop Dis.* 2018;12(10):e0006862.
7. Holm S. A Simple Sequentially Rejective Multiple Test Procedure. *Scand J Stat.* 1979;6(2):65-70.
